# Is There a Potential Market for A2 Milk? Consumer Perception of Dairy Production and Consumption

**DOI:** 10.3390/foods14152567

**Published:** 2025-07-22

**Authors:** Carmen L. Manuelian, Xavier Such, Bibiana Juan, María J. Milán

**Affiliations:** 1 Ruminant Research Group (G2R), Department of Animal and Food Sciences, Universitat Autònoma de Barcelona (UAB), 08193 Bellaterra, Spain; carmen.manuelian@uab.cat (C.L.M.); xavier.such@uab.cat (X.S.); 2 Centre d’Innovació, Recerca i Transferència en Tecnologia dels Aliments (CIRTTA), Department of Animal and Food Sciences, Universitat Autònoma de Barcelona (UAB), 08193 Bellaterra, Spain; bibiana.juan@uab.cat

**Keywords:** A2 milk, consumer attitude, food preference, survey, dairy products, linear multiple regression, sociodemography, milk quality and safety

## Abstract

This online survey aimed to gather consumer opinions on dairy products and production and to identify the potential market for A2 milk (milk containing exclusively β-casein A2, which reduces gastrointestinal discomfort after consumption). The questionnaire included seven sections covering the consumption of dairy products, sociodemographic aspects, awareness and purchase intention of A2 milk, questions about milk as a source of nutrients and health benefits, the environmental impact of milk production, and alternatives to cow milk. Responses from 672 Spanish consumers categorized into clusters (according to their milk consumption and their discomfort after drinking it), gender, age, educational level, and milk taste preference were analyzed using a linear multiple regression model. Dairy consumers not experiencing discomfort after drinking milk (62.6%) and those who preferred the taste of milk over plant-based alternatives (64.0%) demonstrated better knowledge of milk nutrients and its health benefits. Participants’ age, gender, and education level also influenced their perceptions, with older participants, women, and those with university education generally showing better results. In conclusion, clusters impact consumers’ milk perceptions as a nutritional source and its health benefits. The positive perception of milk’s nutritional benefits among dairy consumers experiencing discomfort after drinking milk (17.3%) positions them as a strong target market for A2 milk.

## 1. Introduction

Western countries’ dairy sector has recently undergone profound transformations that reflect consumer preferences and broader societal trends. Over the past decades, a steady decrease in fluid milk consumption has been observed in several countries [1,2], while dairy products, such as cheese, yogurt, and other fermented products, have increased [2]. Interrelated factors underpin this shift in consumption. Firstly, the growing public awareness of nutrition and health has led consumers to re-evaluate their dietary choices, often prioritizing products perceived as healthier [3,4,5]. Secondly, environmental concerns have become increasingly important as consumers seek to minimize their ecological footprint and practice more sustainable consumption (e.g., GMO-free, organic) [6]. Furthermore, ethical and social aspects related to production systems (e.g., animal welfare and local production) have also influenced purchasing decisions [7,8,9]. Some of these factors are expected to have a greater impact on shaping consumer preferences in the future. For example, younger consumers are expected to choose dairy products with lower fat and sugar contents or products that address food intolerances and allergies [10,11]. At the same time, lifestyle choices and the health requirements of an aging population are likely to increase demand for fortified (e.g., with vitamins and minerals) and functional (e.g., addressing specific nutritional needs) products [10,11]. To address these changing dynamics and cope with the growing interest in plant-based milk alternatives, the dairy industry has proactively expanded its market, offering a wide range of dairy products such as semi-skimmed, skimmed, lactose-free, and Ca-fortified and omega-3-fortified variants.

In recent years, several studies compared dairy and plant-based milk substitute consumption and preference to understand consumer behavior toward dairy products, revealing that milk consumption strength is related to consumers’ habitude to drinking it, taste and flavor likeness [12,13,14], and positive emotions associated with it (e.g., happy, positive, good) [12]. Consumers still have the misconception that milk is high in fat, cholesterol, and calories [12]. However, they believe in its nutritional benefits, expect to be healthy, and appreciate organic, reduced fat, probiotics, and vitamin-fortified milk with digestive benefits [12]. On the other hand, the increase in plant-based beverage consumption could be related to the environmental impact of milk production and animal welfare [12,15]. However, price, sensory properties, and familiarity are perceived as barriers to plant-based beverage consumption [15,16,17].

In this context, A2 milk has emerged as a functional dairy food based on its natural health benefits compared to conventional milk [18]. This milk differs from conventional milk in the amino acid sequence of one of its proteins. At position 67 of the β-casein peptide chain, A2 milk presents the amino acid proline instead of histidine, which prevents the release of larger amounts of the peptide β-casomorphin-7 (BCM-7) during milk digestion reducing gastrointestinal symptoms [19,20,21,22]. Therefore, A2 milk is a promising alternative for consumers who, without being allergic or lactose intolerant, experience gastrointestinal discomfort after consuming milk. A2 milk was first commercialized by the A2 Milk Company in 2000 in New Zealand, which has marketed premium A2 milk and dairy products in New Zealand, the UK, the USA, China, and Australia [23]. Consumers’ interest in health, improving digestibility, and meeting specific dietary needs, such as for children, the elderly, and intolerant people, have driven the market growth of A2 dairy products worldwide [24,25]. Moreover, consumers are willing to pay a EUR 0.20 premium price for A2 milk compared with fresh lactose-free milk [24].

We hypothesized that the factors that most influence milk consumption are the perception of the product and its production, the discomfort produced after being consumed, and better organoleptic characteristics than the substitutes. Thus, this study aimed to gather consumers’ opinions about dairy products and their production and to identify a potential market for A2 milk in Spain. Ultimately, we seek to provide information to help the dairy sector establish future strategies for market adaptation and product innovation, such as A2 milk.

## 2. Materials and Methods

### 2.1. Ethical Statement

**Figure 1:**
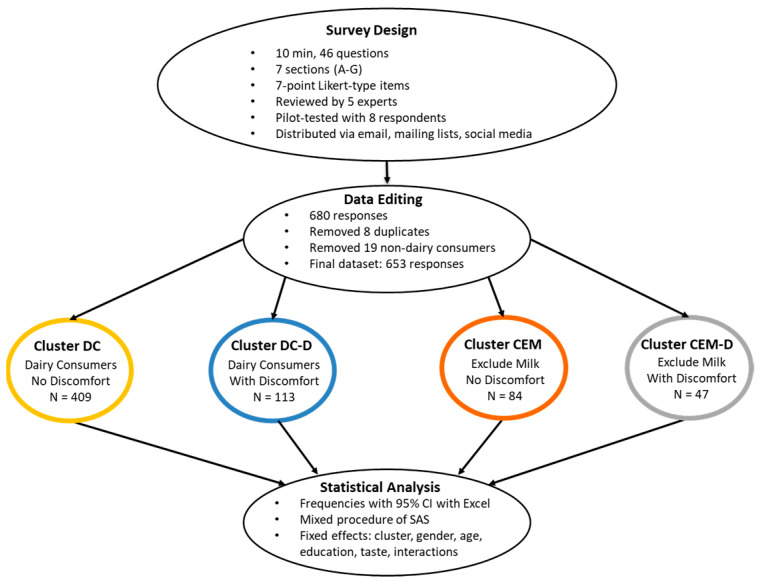
Schematic diagram of the methodological approach followed.

Survey participation was voluntary, completely anonymous, and in agreement with the Declaration of Helsinki version 2013 for research involving human subjects from the World Medical Association (WMA, 2023). Data were processed under the General Data Protection Regulation 2016/679 (GDPR) and the Data Protection Act 2018. The online questionnaire addressed to adults included a cover letter explaining the survey study and requested the acceptance of the informed consent statement before starting. Participants could withdraw from the survey at any time before the end of the questionnaire. Because sensitive data were minimized in the questionnaire to preserve anonymity, participants’ identities were unknown, and the completed questionnaire withdrawal was not possible. A schematic overview of the methodology flow followed is shown in Figure 1.

### 2.2. Survey Design

The questionnaire lasted 10 min and consisted of 46 questions (Q) divided into 7 sections: (A) dairy product consumption; (B) opinion on milk as a source of nutrients based on a 5-point Likert-type item (where 1 means “not at all” and 5 means “very much”) or “I do not know”; (C) opinion on milk and health (5-point Likert-type item or “I do not know”); (D) opinion on milk production and environmental impact (5-point Likert-type item or “I do not know”); (E) opinion on alternatives to cow milk (5-point Likert-type item); (F) opinion on A2 milk benefits and intention to purchase; and (G) sociodemographic information. The questionnaire was in Spanish (English translation is displayed in Table 1). The questionnaire’s development followed the workflow described by Manuelian et al. [26]. It was drafted based on Dillman’s [27] structure and wording recommendations and reviewed by 5 researchers in Food and Animal Science from the Universitat Autònoma de Barcelona (Spain). With the suggested amendments, the questionnaire was implemented in the Google Forms platform and pilot-tested with 8 respondents covering different sociodemographics to improve questionnaire clarity, accuracy, and general intelligibility. The final version was shared via e-mail, mailing lists, and social media platforms during 2 mo from 28 March to 28 May 2023.

### 2.3. Data Editing

A total of 680 questionnaires were completed, but 4 pairs of potential duplicates were identified based on sociodemographic questions and specific questions less prone to change over time. All those potential duplicates were from dairy consumers who did not feel discomfort after drinking milk—which was the most numerous group—and were eliminated (*n* = 8). Non-dairy consumers (*n* = 19) were also discarded. The final dataset included 653 questionnaires grouped into 4 clusters as follows: (i) dairy consumers not feeling discomfort after drinking milk (DC; *n* = 409); (ii) dairy consumers feeling discomfort after drinking milk (DC-D; *n* = 113); (iii) dairy consumers excluding milk not feeling discomfort after drinking milk (CEM; *n* = 84); and (iv) dairy consumers excluding milk feeling discomfort after drinking milk (CEM-D; *n* = 47).

### 2.4. Statistical Analysis

Analysis was performed with SAS v9.4 (SAS Inst. Inc., Cary, NC, USA) and Microsoft Excel 2016 (Microsoft Office Professional Plus, Microsoft Corporation, Albuquerque, NM, USA). Qualitative questions were evaluated based on relative frequencies with a 95% Confidence Interval (CI). Sources of variation for Likert-scale type questions were evaluated through a MIXED procedure of SAS after confirming the normality of the residuals according to the following model:

y_ijklmno_ = µ + Cluster_i_ + Gender_j_ + Age_k_ + Education_l_ + Taste_m_ + (Cluster × Gender)_ij_ + (Cluster × Age)_ik_ + (Cluster × Education)_il_ + (Cluster × Taste)_im_ + Participant_n_ + e_ijklmno_ (1)

where y_ijklmno_ is the dependent variable; µ is the overall intercept of the model; Cluster_i_ is the fixed effect of the ith participant cluster (4 classes: DC, DC-D, CEM, CEM-D); Gender_j_ is the fixed effect of the *j*th gender of the respondents (2 classes: woman, including non-binary and prefer not to respond; man); Age_k_ is the fixed effect of the *k*th age class of the respondents (4 categories: between 18 and 35 years old; between 36 and 45 years old; between 46 and 55 years old; ≥56 years old); Education_l_ is the fixed effect of the *l*th education class of the respondents (2 categories: university; non-academic, who has completed primary or secondary education); Taste_m_ is the fixed effect of the *m*th taste preference of the respondents (2 categories: like milk better than plant-based alternatives; do not like milk better than plant-based alternatives); (Cluster × Gender)_ij_ is the fixed interaction effect between cluster and gender; (Cluster × Age)_ik_ is the fixed interaction effect between cluster and age; (Cluster × Education)_il_ is the fixed interaction effect between cluster and education; (Cluster × Taste)_im_ is the fixed interaction effect between cluster and taste; Participant_n_ is the random effect of the *n*th participant (*n* = 1 to 653) ~N(0, σ^2^_PARTICIPANT_); and e_ijklmno_ is the random residual ~N(0, σ^2^_e_), where σ^2^_e_ is the residual variance. Least squares means multiple comparisons were performed applying Tukey’s post hoc test, and significance was established at *p* < 0.05.

## 3. Results

### 3.1. Participant Characteristics and Self-Defined Dairy Product Consumption

Most participants were included in cluster DC (62.6%; 95% CI, 58.9–66.3%), whereas Cluster DC-D (17.3%; 95% CI, 14.4–20.2%) and CEM (12.9%; 95% CI, 10.3–15.4%) were equally represented. In contrast, cluster CEM-D was less represented (7.2%; 95% CI, 5.2–9.2%). Therefore, 21.6% of dairy product consumers reported gastrointestinal discomfort after milk consumption (DC and DC-D). Among consumers excluding fluid milk, 35.9% reported that they had stopped consuming it due to gastric discomfort (CEM and CEM-D). In all four clusters, most participants were women aged 26 to 65 years old and had university studies (Table 2). Participants mainly lived in the Spanish regions of Catalonia (39.9%), Navarra (16.8%), Valencia (7.6%), and Galicia (7.0%).

The frequency of consumption of dairy products and likeness to plant-based alternatives differed across clusters. The majority of cluster DC consumes milk daily (87.3%), cluster DC-D consumes milk more than twice a week (92.0%), and clusters CEM and CEM-D consume dairy products more than once a week (>96%). Moreover, only cluster DC preferred milk (77.8%; 95% CI, 73.7–81.8%), whereas clusters CEM (77.4%; 95% CI, 68.4–13.7%) and CEM-D (68.1%; 95% CI, 54.8–81.4%) preferred plant-based beverages and have substituted milk with plant-based beverages (CEM, 57.1%; CEM-D, 72.3%).

### 3.2. Participants’ Uncertainty on 5-Point Likert Scale Questions

Table 3 displays the proportion of participants indicating ‘I don’t know’ in Sections B, C, and D of the questionnaire. In all three sections, the proportion was similar across clusters. In Section B, which was related to milk nutrients, Q_B1_, Q_B2_, and Q_B5_ had the highest proportion of ‘I don’t know’. In Section C, which relates milk consumption to health, and Section D, regarding the environmental impact of milk production, the degree of uncertainty was higher than from questions in Section B, reaching up to 38.3%. Nevertheless, participants have a clear opinion on the need to make efforts to improve animals’ welfare (Q_D3_).

### 3.3. Opinion on Milk as a Source of Nutrients

**Figure 2:**
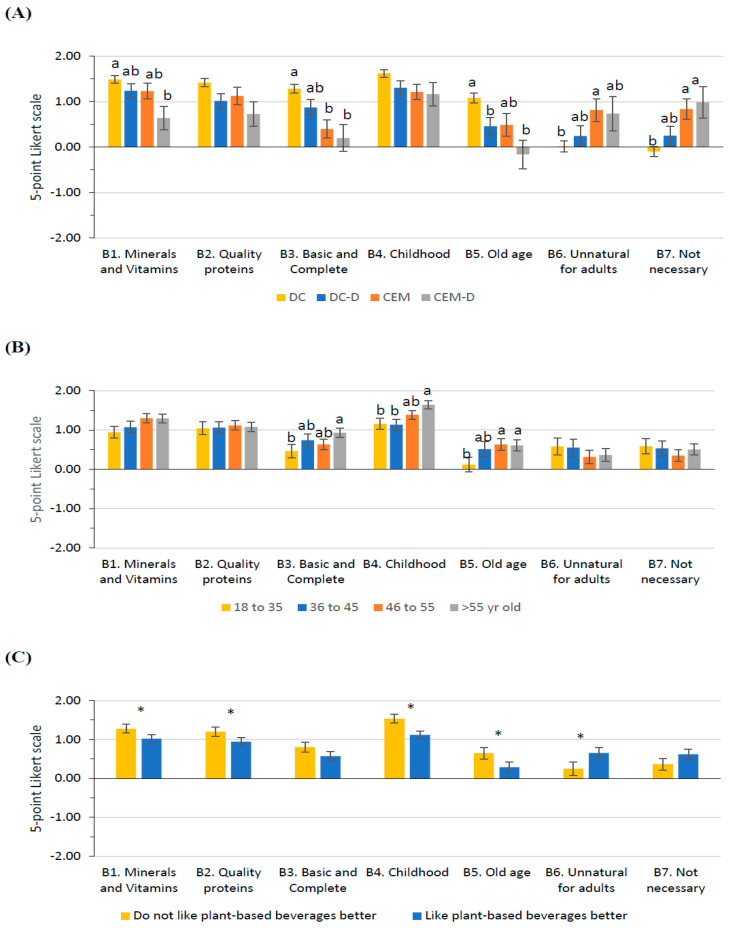
Least square means (with SE) for Section B: Opinion on milk as a source of nutrients. (**A**) Cluster (DC, *n* = 409; DC-D, *n* = 113; CEM, *n* = 84; CEM-D, *n* = 47), (**B**) Age class (18–35 years old, *n* = 130; 36–45 years old, *n* = 99; 46–55 years old, *n* = 209; >55 years old, *n* = 215), and (**C**) (Do not like plant-based beverages better, *n* = 418; Like plant-based beverages better, *n* = 235). See Table 1 for the complete questions. Different superscript or * within the question indicates significant differences (*p* < 0.05) based on Tukey’s post hoc test.

Cluster significantly impacted participants’ opinions in almost all questions about milk nutrients (Figure 2A). Participants from cluster DC reported a better knowledge of milk nutrients and the relevance of consuming milk than those from cluster CEM-D. Participants from cluster DC strongly agreed that milk is a relevant source of Ca, P, Mg, and vitamins (Q_B1_), whereas cluster CEM-D indicated a more neutral position. For Q_B2_, even if the cluster single effect was not significant, there was a significant interaction between the cluster and the taste preference. Among those who like plant-based beverages more than milk, cluster DC (1.42 ± 0.09) agreed more with milk containing high-quality proteins than cluster CEM (0.66 ± 0.19; *p* = 0.016). Moreover, within cluster CEM, those who like milk better agreed more with that statement (Like milk better, 1.60 ± 0.27; *p* = 0.017). For Q_B3_, cluster CD agreed more strongly that milk is an essential, complete, and relatively cheap food than those who do not regularly consume it (CEM and CEM-D). Still, there was a significant interaction with the level of studies. For those participants with non-academic studies, cluster DC reported a strong agreement with this statement (1.32 ± 0.16), whereas clusters CEM (0.01 ± 0.32; *p* = 0.007) and CEM-D (−0.38 ± 0.50; *p* = 0.030) indicated neutrality and disagreement, respectively. For Q_B4_, even if the cluster single effect was not significant, there was a significant interaction between cluster and age. Within cluster CEM, younger participants (18 to 35 years old, 0.72 ± 0.24; 36 to 45 years old, 0.45 ± 0.32) were less aware (*p* < 0.05) that milk is an essential food during childhood than older participants (46 to 55 years old, 1.89 ± 0.22; >55 years old, 1.80 ± 0.21). For Q_B5_, even if cluster DC was more aware that milk is an essential food during old age than clusters CEM-D and DC-D, some differences in gender perception were detected. Women from cluster CD agreed with considering milk as a necessary food during old age, whereas those in clusters CEM (0.00 ± 0.25; *p* = 0.004) and CEM-D (−0.13 ± 0.29; *p* = 0.004) had a more neutral position. For Q_B6_, cluster CEM was more in agreement than DC, with adults not needing milk in adulthood. However, there was an interaction with age, with older participants from cluster DC (46 to 55 years old, −0.28 ± 0.16; >55 years old, −0.13 ± 0.14) being more in disagreement with this statement than younger participants in this cluster (18 to 35 years old, 0.60 ± 0.20; *p* < 0.05). For Q_B7_, cluster DC disagreed more than clusters CEM and CEM-D about milk not being necessary because the same nutrients can easily be obtained from other foods.

Participants’ age also modified their perceptions for Q_B3_, Q_B4_, and Q_B5_ (Figure 2B). Older participants are more in agreement with milk being a basic food, very complete, and relatively cheap (Q_B3_) and that milk is an essential food during old age (Q_B5_) compared with younger participants. Concerning Q_B4_, although the agreement with milk as a necessary food in childhood increased with age, there was a significant interaction between age and cluster, as previously described.

The level of studies also impacted participants’ opinions of Q_B1_, Q_B2_, and Q_B3_. Participants with university degrees were more aware (1.34 ± 0.15) that milk is an important source of Ca, P, Mg, and vitamins (Q_B1_; University, 1.34 ± 0.15; Non-academic, 0.96 ± 0.07); contains high-quality proteins (Q_B2_; University, 1.24 ± 0.07; Non-academic, 0.90 ± 0.16); and is an essential food, very complete, and relatively cheap (Q_B3_; University, 0.92 ± 0.08; Non-academic, 0.47 ± 0.17) than those with non-academic studies. However, a significant interaction between the level of studies and cluster was detected for Q_B3_, as described before. Moreover, women were more in agreement than men that we could easily get from other foods the same nutrients as milk (Women, 0.70 ± 0.12; Men, 0.29 ± 0.17; *p* = 0.015).

Taste preference also modified participants’ perceptions in Q_B1_, Q_B2_, Q_B4_, Q_B5_, and Q_B6_ (Figure 2C). Those who did not like plant-based beverages better than milk were more aware that milk is an important source of Ca, P, Mg, and vitamins (Q_B1_); is an essential food during childhood (Q_B4_) and adulthood (Q_B5_); that milk consumption is still relevant during adulthood (Q_B6_); and that it contains high-quality proteins (Q_B2_) than those who preferred plant-based beverages. However, as previously mentioned, there was a significant interaction between the cluster and the taste preference for Q_B2_.

### 3.4. Opinion on Milk and Health

**Figure 3:**
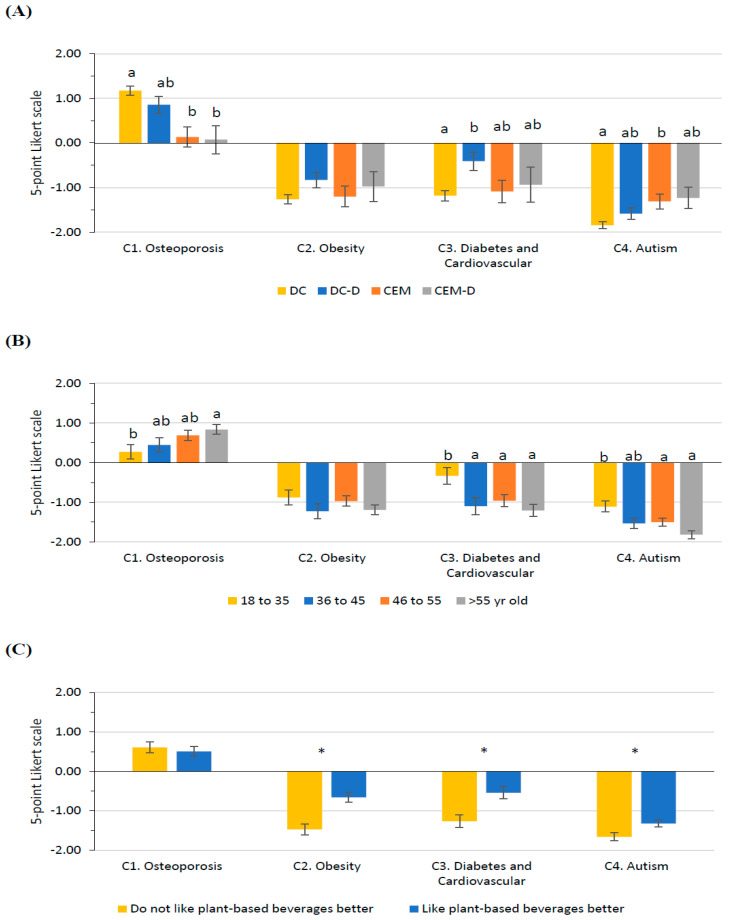
Least-square means (with SE) for Section C: Opinion on milk and health. (**A**) Cluster (DC, *n* = 409; DC-D, *n* = 113; CEM, *n* = 84; CEM-D, *n* = 47), (**B**) Age class (18–35 years old, *n* = 130; 36–45 years old, *n* = 99; 46–55 years old, *n* = 209; >55 years old, *n* = 215) and (**C**) (Do not like plant-based beverages better, *n* = 418; Like plant-based beverages better, *n* = 235). See Table 1 for the complete questions. Different superscript or * within the question indicates significant differences (*p* < 0.05) based on Tukey’s post hoc test.

Cluster impacted participants’ opinions in almost all questions related to the relevance of milk consumption on health (Figure 3A). For Q_C1_, cluster DC was more aware that milk is a key food in preventing osteoporosis, especially compared with clusters CEM and CEM-D. However, there was an interaction with the level of studies. Those with university studies in cluster CEM agreed more (0.68 ± 0.19) with this statement than those with non-university studies (−0.41 ± 0.36; *p* = 0.039). Moreover, participants from cluster DC with non-academic studies agreed (1.20 ± 0.17) that milk is a key food in preventing osteoporosis, whereas participants from cluster CEM with non-academic studies disagreed (−0.41 ± 0.36; *p* = 0.002). Gender also modified Q_C1_ clusters’ opinion. Women in clusters DC (1.19 ± 0.10) and DC-D (0.97 ± 0.20) had a better perception of milk as a key food in the prevention of osteoporosis than those in clusters CEM (−0.30 ± 0.24) and CEM-D (−0.15 ± 0.30; *p* < 0.05).

All clusters disagreed with milk consumption being related to a higher risk of obesity (Q_C2_), diseases such as diabetes or cardiovascular problems (Q_C3_), and autism (Q_C4_). Related to the higher risk of obesity (Q_C2_), the disagreement was stronger for those who did not like plant-based beverages better than milk for clusters DC-D (Do not like plant-based beverages better, −1.16 ± 0.19; Like plant-based beverages better, −0.50 ± 0.21; *p* = 0.043) and CEM (Do not like plant-based beverages better, −1.84 ± 0.31; Like plant-based beverages better, −0.56 ± 0.24; *p* = 0.001). Regarding the increased risk of diseases such as diabetes or cardiovascular problems (Q_C3_), there was also a stronger disagreement among those who did not like plant-based beverages better than milk for clusters DC (Do not like plant-based beverages better, −1.44 ± 0.11; Like plant-based beverages better, −0.92 ± 0.15; *p* = 0.048) and CEM (Do not like plant-based beverages better, −1.76 ± 0.34; Like plant-based beverages better, −0.41 ± 0.27; *p* = 0.002). Regarding the increased risk of autism in children (Q_C4_), a stronger disagreement was observed for those who did not like plant-based beverages better than milk for CEM-D (Do not like plant-based beverages better, −1.72 ± 0.31; Like plant-based beverages better, −0.74 ± 0.25; *p* = 0.024). In CEM-D, that disagreement was even stronger for older (55 years old, −2.0 ± 0.27) than younger participants (18 to 35 years old, −0.27 ± 0.46; 46 to 55 years old, −1.16 ± 0.29: *p* < 0.05). In addition, among younger participants, those belonging to cluster DC (−1.85 ± 0.12) disagreed much more with this statement than those in cluster CEM (−0.92 ± 0.23; *p* = 0.026). Moreover, among participants who preferred the taste of plant-based alternatives, those from the DC cluster (−1.79 ± 0.10) disagreed more strongly with this statement than those from cluster CEM-D (*p* = 0.003).

Participants’ age modified participants’ perceptions regarding the relationship between milk consumption and health (Figure 3B). As age increased, participants were more in agreement with milk being a key food in preventing osteoporosis (Q_C1_) and more in disagreement with milk consumption being related to a greater risk of certain diseases such as diabetes or cardiovascular problems in adulthood (Q_C3_) and to an increased risk of autism in children (Q_C4_). However, as reported before, an interaction with the cluster was observed for Q_C4_.

Taste preference modified participants’ perceptions of Q_C2_, Q_C3_, and Q_C4_ (Figure 3C). Those who did not like plant-based beverages more than milk were more in disagreement with milk consumption being associated with some diseases. Nevertheless, as mentioned above, there was a significant interaction between cluster and taste preference for all these statements.

### 3.5. Opinion on Milk Production and Environmental Impact

**Figure 4:**
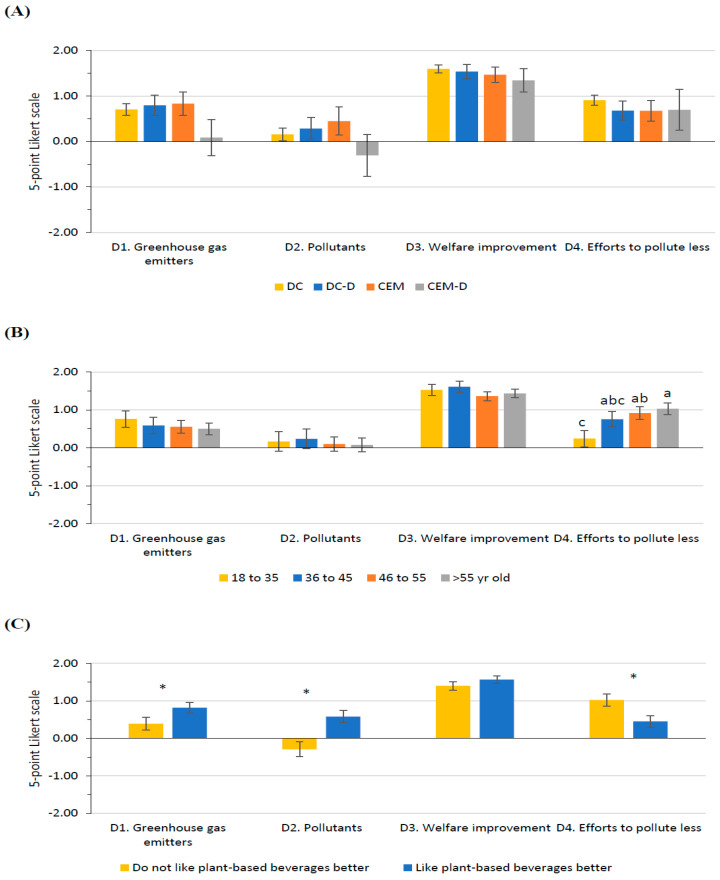
Least-square means (with SE) for Section D: Opinion on milk production and environmental impact. (**A**) Cluster (DC, *n* = 409; DC-D, *n* = 113; CEM, *n* = 84; CEM-D, *n* = 47), (**B**) Age class (18–35 years old, *n* = 130; 36–45 years old, *n* = 99; 46–55 years old, *n* = 209; >55 years old, *n* = 215), and (**C**) (Do not like plant-based beverages better, *n* = 418; Like plant-based beverages better, *n* = 235). Different superscript or * within the question indicates significant differences (*p* < 0.05) based on Tukey’s post hoc test.

The cluster (Figure 4A), study level, and gender did not modify participants’ perception of the environmental impact of milk production. Participants had a more neutral opinion on dairy farms being the largest emitters of greenhouse gases (Q_D1_), slightly disagreed that dairy farms pollute the environment more than most crops used to produce plant-based beverages (Q_D2_), agreed with farmers trying to pollute less and be more sustainable (Q_D4_), and believe that it is important that farms improve the animals’ welfare (Q_D3_). However, participants’ perception of the effort of farmers to pollute less and be more sustainable (Q_D4_) was enhanced with age, from a more neutral position to more in agreement with the statement (Figure 4B). Moreover, participants reported a more positive perception of milk production when they did not like plant-based beverages better than milk (Figure 4C).

### 3.6. Opinion on Alternatives to Cow Milk

**Figure 5:**
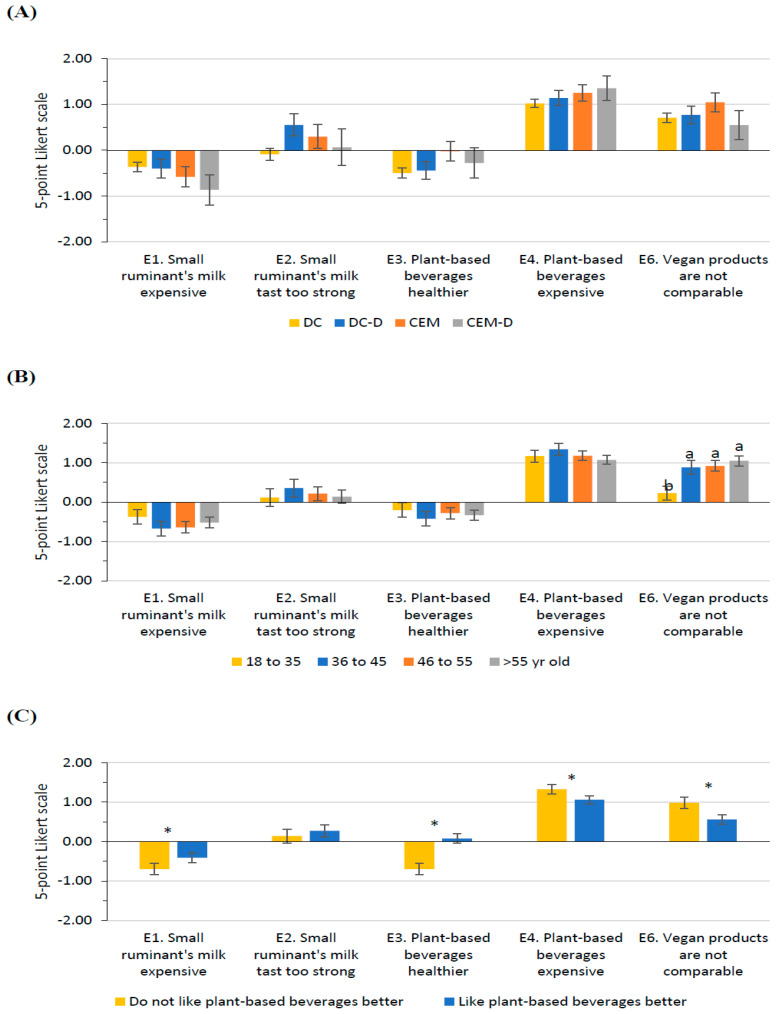
Least-square means (with SE) for Section E: Opinion on alternatives to cow milk. (**A**) Cluster (DC, *n* = 409; DC-D, *n* = 113; CEM, *n* = 84; CEM-D, *n* = 47), (**B**) Age class (18–35 years old, *n* = 130; 36–45 years old, *n* = 99; 46–55 years old, *n* = 209; > 55 years old, *n* = 215), and (**C**) (Do not like plant-based beverages better, *n* = 418; Like plant-based beverages better, *n* = 235). Different superscript or * within the question indicates significant differences (*p* < 0.05) based on Tukey’s post hoc test.

Cluster (Figure 5A) and studies level did not modify participants’ perception of alternatives to cow milk, such as other ruminant species producing milk or plant-based alternatives. Nevertheless, age (Figure 5B), taste preferences (Figure 5C), and gender modified their perception. Participants over 36 considered that vegan products that replace dairy products are not comparable and that nothing compares to a good cheese or yogurt (Q_E6_; Figure 5B). Those who do not like plant-based beverages better than milk disagreed more with sheep and goat milk not being an alternative to cow milk since they are more expensive (Q_E1_) and with plant-based beverages being healthier than animal-based ones (Q_E3_). Moreover, they agreed that plant-based beverages are more expensive than milk (Q_E4_) and that vegan products that replace dairy products are not comparable (Q_E6_). Gender also modified participants’ opinions. Women disagreed more than men with sheep and goat milk not being an alternative to cow milk since they are more expensive (Q_E1_; Women, −0.73 ± 0.11; Men, −0.37 ± 0.16; *p* = 0.029). Women also agreed more that plant-based beverages are more expensive than milk than men (Q_E4_; Women, 1.42 ± 0.09; Men, 0.96 ± 0.13; *p* < 0.001).

### 3.7. Opinion on A2 Milk Benefits and Intention to Purchase

Most participants in all four clusters were unaware of A2 milk’s existence on the market (>74.5%; Table 4). Those who have heard about this milk associated it with being healthier, particularly those from cluster CEM-D (19.1%; Table 4). Moreover, about 4.0% of participants from clusters DC and CEM-D thought that A2 milk would be more expensive than regular milk (Table 4).

Considering A2 milk’s gastrointestinal benefits, more than half of the cluster DC-D participants and about one-third of the cluster CEM-D participants thought this product could interest them (Table 4). However, a high proportion of uncertainty is also present in all four clusters. About half of the participants from clusters DC, CEM, and CEM-D thought that A2 milk was not relevant to them, whereas 44.2% of participants from cluster DC-D would buy it at the same price as regular milk (Table 4). Moreover, about one-third of consumers with discomfort after consuming milk would buy A2 milk if it was above 10% more expensive than regular milk (DC-D, 37.2%; CEM-D, 31.9%; Table 4).

## 4. Discussion

**Figure 6:**
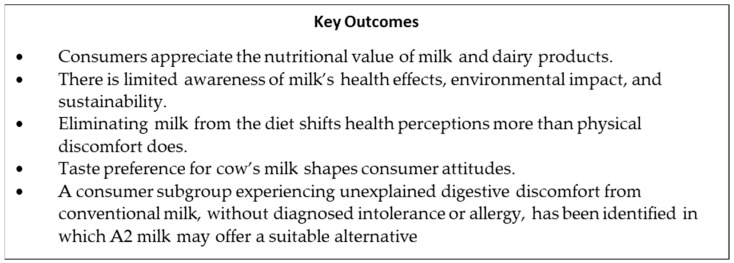
Main findings of this study.

This study focused on the perception of dairy consumers regarding milk’s nutritional characteristics, its impact on human health, aspects related to its production, and substitutes for cow milk. A summary of the key results of this study is shown in Figure 6.

**Figure 7:**
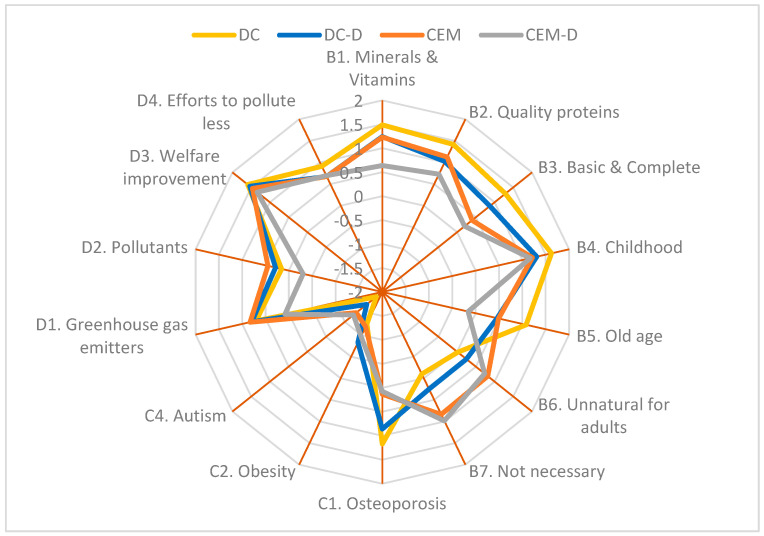
Overview of consumers’ opinion on milk as a source of nutrients and health benefits, and the environmental impact of milk production by cluster (DC, *n* = 409; DC-D, *n* = 113; CEM, *n* = 84; CEM-D, *n* = 47) based on a 5-point Likert scale. See Table 1 for complete questions.

**Figure 8:**
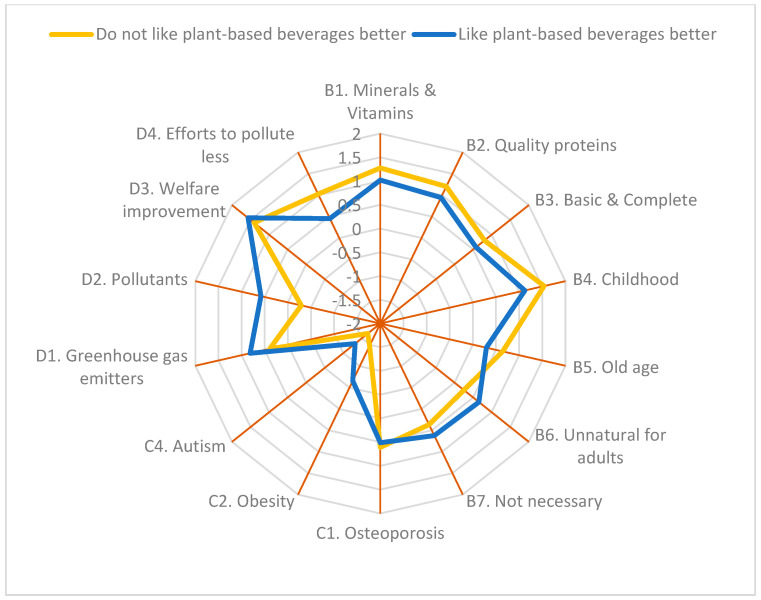
Overview of consumers’ opinion on milk as a source of nutrients and health benefits, and the environmental impact of milk production by preference for milk taste over plant-based beverages based on a 5-point Likert scale. See Table 1 for complete questions.

As summarized in Figure 7 and Figure 8, Cluster DC and those who did not prefer the plant-based alternatives’ taste over milk reported a better knowledge of milk nutrients and the benefits of milk consumption on health than cluster CEM-D or those who preferred plant-based alternatives over milk. Most participants in all four clusters were unaware of the existence of A2 milk, and similar to Bentivoglio et al. [24], >75% of cluster DC-D would buy it up to 20% more expensive than regular milk. Recognition of the nutritional value of milk, its health benefits, and the acceptability of production systems are three very important aspects in assessing whether A2 milk will be acceptable in the marketplace.

### 4.1. Consumer Behavior Towards Dairy Products

Consumers’ behavior in Western countries is complex due to the access to a wide range of products [28], and their perception and attitude towards a product are key factors in their consumption decisions [12,29,30,31]. Different factors condition consumers’ perceptions and attitudes towards a product such as lifestyle [32], social environment [33], and sociodemographic or personal factors (e.g., age, education) [34,35,36,37]. However, to the best of our knowledge, the present study is the first to demonstrate that gastrointestinal discomfort after milk consumption (without being milk intolerant or allergic) and taste preference for plant-based beverages or milk influenced the population’s perception of milk. We consider these aspects essential in understanding the role that a product such as A2 milk can play in the market. Therefore, our findings contribute to a more comprehensive understanding of the determinants of dairy consumption and provide valuable insights for both industry stakeholders and public health initiatives aimed at promoting informed consumer choices.

### 4.2. Consumer Perception of Milk Nutritional Traits, Health Benefits, and Production

Our results revealed that consumers’ perceptions of the nutritional characteristics of milk and the importance of its consumption at different stages of life, especially in childhood, were very positive (Figure 7). This finding aligns with previous studies conducted in North Carolina [12], France and Portugal [33], and Poland [5] on dairy products and plant-based beverages.

In line with Haas et al. and Vargas-Bello-Pérez et al. [16,38], consumers in the present study, particularly those groups who have not excluded liquid milk from their diet, agreed that milk consumption has beneficial effects on human health, especially in preventing osteoporosis. Moreover, consumers expressed skepticism toward certain controversial and inconsistent studies that associate milk consumption with adverse health effects such as autism and obesity (Figure 7) [39,40]. However, these questions presented a high number of “I don’t know” responses, suggesting a lack of awareness or understanding of the topic. This highlights the need for clear and evidence-based communication from experts, healthcare professionals, and other reliable sources to address misconceptions and provide accurate information to consumers [41].

In the present study, despite respondents agreeing that dairy farms are significant emitters of greenhouse gases, they did not perceive them as more polluting than most crops used for producing plant-based beverages and considered that farmers are making efforts to reduce pollution and adopt more sustainable practices. Nevertheless, these two statements also obtained a high number of “I don’t know” responses, which underscores the need for the dairy sector to improve communication in its sustainability initiatives [42].

Similar to other studies [43,44], consumers believed that it is very important that farmers make efforts to improve animal welfare, as those authors observed that nutritional and animal welfare aspects were more important to consumers than the environmental sustainability of production (Figure 7). In the present study, there was a high consensus across clusters, age, level of education, and whether they like milk more than plant-based beverages concerning animal welfare. These results agreed with the Eurobarometer, which shows that most European citizens consider it very important to protect the welfare of farm animals [45]. Moreover, it highlights one of the key factors influencing competition between animal-based and plant-based products [46,47,48].

### 4.3. Age and Taste Modified Milk Perception

Consumer age influenced the perceptions of milk’s nutritional value and its effects on human health. Younger consumers were less likely than older consumers to agree that milk is a relatively inexpensive staple food, essential during childhood and old age, and important in preventing osteoporosis. Although younger consumers disagreed with the idea that milk consumption may be linked to certain health problems, their level of disagreement was lower than that of older consumers, which suggests a lack of basic knowledge among younger generations regarding the nutritional composition of dairy products [13]. However, they may also reflect a broader trend observed in other studies, which could have significant implications for the future consumption of dairy products. Since younger generations represent the future consumers and are the primary decision-makers in their children’s diets, their attitudes toward dairy could play a decisive role in shaping market trends [13]. Previous research [49] has demonstrated that familiarity with food products is positively associated with sensory preference, having a strong influence on the acceptability of many foods. In particular, Refs. [12,50] report that habit and taste are among the primary drivers of dairy consumption. Consequently, declining dairy consumption during childhood could have long-term effects on future consumption patterns [13,51].

As reported by several authors, taste is one of the most influential sensory attributes in milk consumption [34,52,53]. In the present study, we also observed that consumers who preferred plant-based beverages’ taste over milk had a more negative perception of dairy products in terms of their nutritional value, health effects, and environmental impact (Figure 8). These findings align with previous studies in which perceptions and motivations were related to sensory expectations and show that the experience of positive emotions related to the consumption of food products is among the most important drivers in the acceptance and choice of these products [14,17,54]. Our results, furthermore, show that younger consumers give a significantly lower score than older ones to the statement indicating that vegan products are not well-accepted and that nothing is comparable to a good cheese or yogurt, which reinforces the aforementioned idea of the importance of milk and dairy product consumption during childhood.

### 4.4. Potential Market for A2 Milk

Our findings indicate that most differences emerge between those who regularly consume milk (clusters DC and DC-D) and those who have excluded milk from their diet (clusters CEM and CEM-D), regardless of whether they report discomfort after consumption, in agreement with previous studies on lactose-free milk, where people who have consumed milk in the past are more prone to purchase lactose-free milk because they have a strong trust toward milk [55]. Consumers who have eliminated milk from their diet, including those who did so due to discomfort, have a more negative perception of dairy products than those who continue consuming it. This suggests that their decision to stop drinking milk may not be solely due to digestive discomfort but could also stem from other underlying reasons. In fact, this group scored highest on the statement that milk consumption is unnecessary because its nutrients can be obtained from other foods. As a result, it seems unlikely that consumers who avoid liquid milk (CEM-D) would consider A2 milk a viable option for reintroducing milk into their diet. However, they may still represent a potential market for dairy products made with A2 milk, as 55.3% of this group indicated a willingness to purchase such products. However, the DC-D cluster presented similarities with the DC cluster in nearly all aspects, except for their views on milk as an essential food in old age and its potential links to cardiovascular diseases and diabetes. This positive perception of milk’s nutritional benefits among DC-D consumers positions them as a strong target market for A2 milk, with only 5.3% dismissing it as a viable option and 81.4% expressing a willingness to purchase it.

### 4.5. Limitations, Applications, and Perspectives

The present survey study provides insight into dairy consumers’ perception of milk’s nutritional value, its impact on human health, milk production, and substitutes for cow milk. It also assessed the intention to purchase a new dairy product, namely, A2 milk. However, this study may not entirely reflect the actual demographics in Spain because we used a voluntary survey methodology. Nevertheless, voluntary surveys are still considered a relevant tool to gather information at the population level [56]. In the present study, women and consumers with university studies were overrepresented compared to the general Spanish population because the questionnaire was distributed primarily through academic channels. This bias may have influenced the positive perception consumers had on the nutritional characteristics and health benefits of milk, as well as the low trust consumers placed in milk myths or poorly verified news. However, we believe this bias minimally impacted the two key aspects of this study, which are feeling discomfort after drinking milk and whether consumers prefer the taste of milk to plant-based beverages. Furthermore, the consistency of our results with previous studies in other regions lends validity to our findings.

Given the well-established link between familiarity, acceptability, and sensory expectations, A2 milk could be an appealing alternative for consumers who experience digestive discomfort after consuming regular milk but still value the sensory qualities of dairy products. In this context, marketing strategies for A2 milk should primarily target these consumers, as well as the parents of young children, by emphasizing its nutritional benefits, particularly during early childhood, while also highlighting its taste and other sensory attributes associated with dairy consumption. Further studies should include the focus group methodology on milk consumers who experience digestive discomfort after consuming regular milk without being lactose intolerant or allergic to milk protein to understand better the intention to purchase.

## 5. Conclusions

Milk and dairy product consumers appreciate their nutritional properties but lack knowledge regarding certain health effects associated with their consumption, their environmental impact, and sustainability. However, there is a strong concern for improving animal welfare. The exclusion of liquid milk from the diet had more of an impact on consumers’ perception of milk as a nutritional source and its relation to health than feeling discomfort after consuming milk. Additionally, preference for the taste of cow milk over plant-based alternatives played a significant role in shaping consumer perceptions. Given the widespread lack of consumer awareness regarding A2 milk, the dairy industry must undertake substantial efforts to promote its benefits for a specific population group. Marketing strategies for A2 milk should specifically target consumers of milk and dairy products who experience digestive discomfort after consuming milk despite not being lactose intolerant or allergic to milk proteins. Moreover, marketing strategies should emphasize the nutritional benefits of milk, especially during childhood, while also highlighting its taste and other sensory expectations related to dairy product consumption.

## Figures and Tables

**Table 1:** Questionnaire.

Questions	Choices
Section A: Dairy product consumption	
Q_A1_: Do you consume dairy products?	Yes, I consume dairy products; Yes, I consume dairy products excluding milk; No
Q_A2_: How often do you consume dairy products?	Rarely (less than once a wk); Sometimes (once a wk); Often (between 2 and 5 times a wk); Always (almost daily)
Q_A3_: Do you agree with this statement? ‘Consuming cow milk sometimes creates discomfort.’	Yes; No
Q_A4_: Why don’t you consume dairy products?	I don’t like it; Drinking milk creates discomfort despite not being diagnosed as allergic or intolerant; I’m lactose intolerant; Other
Q_A5_: Have you substituted milk with plant-based beverages?	Yes; No
Section B: Opinion on milk as a source of nutrients	
Q_B1_: Milk is an important source of calcium, phosphorus, magnesium, and vitamins.	Strongly disagree; Disagree; Neutral; Agree; Strongly agree; I don’t know
Q_B2_: Milk contains high-quality proteins.	Strongly disagree; Disagree; Neutral; Agree; Strongly agree; I don’t know
Q_B3_: Milk is an essential food, very complete, and relatively cheap.	Strongly disagree; Disagree; Neutral; Agree; Strongly agree; I don’t know
Q_B4_: Milk is an essential food during childhood.	Strongly disagree; Disagree; Neutral; Agree; Strongly agree; I don’t know
Q_B5_: Milk is an essential food during old age.	Strongly disagree; Disagree; Neutral; Agree; Strongly agree; I don’t know
Q_B6_: Adults do NOT need to drink milk, in fact, the only mammals that consume milk in adulthood are humans.	1 to 5 Likert-type item (where 1 means “Strongly disagree” and 5 means “Strongly agree”)
Q_B7_: Consuming milk is NOT important, you can easily get the same nutrients with other foods.	1 to 5 Likert-type item (where 1 means “Strongly disagree” and 5 means “Strongly agree”)
Section C: Opinion on milk and health	
Q_C1_: Milk is a key food in the prevention of osteoporosis.	Strongly disagree; Disagree; Neutral; Agree; Strongly agree; I don’t know
Q_C2_: Milk consumption is related to a higher risk of obesity.	Strongly disagree; Disagree; Neutral; Agree; Strongly agree; I don’t know
Q_C3_: In adults, milk consumption is related to a greater risk of certain diseases such as diabetes or cardiovascular problems.	Strongly disagree; Disagree; Neutral; Agree; Strongly agree; I don’t know
Q_C4_: In children, milk consumption is linked to an increased risk of autism.	Strongly disagree; Disagree; Neutral; Agree; Strongly agree; I don’t know
Section D: Opinion on milk production and environmental impact	
Q_D1_: Dairy farms are the largest emitters of greenhouse gases.	Strongly disagree; Disagree; Neutral; Agree; Strongly agree; I don’t know
Q_D2_: Dairy farms pollute the environment more than most crops used to produce plant-based beverages.	Strongly disagree; Disagree; Neutral; Agree; Strongly agree; I don’t know
Q_D3_: I believe that it is important that farmers make efforts to improve the welfare of their animals.	Strongly disagree; Disagree; Neutral; Agree; Strongly agree; I don’t know
Q_D4_: Dairy farms are making great efforts to pollute less and be more sustainable.	Strongly disagree; Disagree; Neutral; Agree; Strongly agree; I don’t know
Section E: Opinion on alternatives to cow milk	
Q_E1_: Sheep and goat milk are NOT alternatives to cow milk, since they are more expensive.	1 to 5 Likert-type item (where 1 means “Strongly disagree” and 5 means “Strongly agree”)
Q_E2_: I DO NOT like sheep and goat milk, they have a very strong flavor.	1 to 5 Likert-type item (where 1 means “Strongly disagree” and 5 means “Strongly agree”)
Q_E3_: Plant-based beverages are healthier than those of animal origin.	1 to 5 Likert-type item (where 1 means “Strongly disagree” and 5 means “Strongly agree”)
Q_E4_: In general, plant-based beverages are more expensive than milk.	1 to 5 Likert-type item (where 1 means “Strongly disagree” and 5 means “Strongly agree”)
Q_E5_: I like the taste of plant-based beverages better than milk.	1 to 5 Likert-type item (where 1 means “Strongly disagree” and 5 means “Strongly agree”)
Q_E6_: Vegan products that replace dairy products are not comparable. Nothing compares to a good cheese or yogurt!	1 to 5 Likert-type item (where 1 means “Strongly disagree” and 5 means “Strongly agree”)
Section F: Opinion on A2 milk benefits and intention to purchase	
Q_F1_: In relation to A2 milk:	Until now, I had never heard of A2 milk; Yes, I had heard about it and I associate it with more natural milk; Yes, I had heard about it and I associate it with healthier milk; Yes, I had heard about it and I associate it with better tasting milk; Yes, I had heard about it and I associate it with more sustainable milk; Yes, I had heard about it and I associate it with more expensive milk; Yes, I had heard about it, although I didn’t know what it was or relate it to anything
Q_F2_: Taking into account its gastrointestinal benefits, do you think that A2 milk could be a good option in your case?	Yes; No; I don’t know
Q_F3_: If you could easily find A2 dairy products in the supermarket, would you buy them?	No, this product in my case is not relevant; Yes, but only if they had the same price as regular milk; Yes, even if they were 10% more expensive than regular milk; Yes, even if they were 20% more expensive than regular milk; I would be willing to pay even more than 20% of what regular milk costs
Section G: sociodemographic information	
Q_G1_: In which Spanish autonomous community do you live?	Close list of Spanish autonomous community
Q_G2_: Gender.	Male; Female; Non-binary; Prefer not to answer
Q_G3_: Age.	18 to 25; 26 to 35; 36 to 45; 46 to 55; 56 to 65; 66 to 75; more than 75 yr old
Q_G4_: What is the highest level of education you have completed?	Primary education; Secondary education; Tertiary education

**Table 2:** Profile of the respondents expressed as relative frequency (RF, %) and range (95% CI).

Cluster ^1^	DC (*n* = 409)		DC-D (*n* = 113)		CEM (*n* = 84)		CEM-D (*n* = 47)	
	RF	95% CI	RF	95% CI	RF	95% CI	RF	95% CI
Gender								
Woman	68.0	63.4–72.5	80.5	73.2–87.8	78.6	69.8–87.3	70.2	57.1–83.3
Man	31.3	26.8–35.8	18.6	11.4–25.8	19.1	10.7–27.4	25.5	13.1–38.0
Non-binary	-	-	-	-	1.2	0–3.5	-	-
Prefer not to respond	0.7	0–1.6	0.9	0–2.6	1.2	0–3.5	4.3	0–10.0
Age class, yr old								
18 to 25	3.2	1.5–4.9	9.7	4.3–15.2	3.6	0–7.5	4.3	0–10.0
26 to 35	13.2	9.9–16.5	18.6	11.4–25.8	25.0	15.7–34.3	10.6	1.8–19.5
36 to 45	15.9	12.3–19.4	13.3	7.0–19.5	13.1	5.9–20.3	17.0	6.3–27.8
46 to 55	32.3	27.7–36.8	35.4	26.6–44.2	26.2	16.8–35.6	31.9	18.6–45.2
56 to 65	31.8	27.3–36.3	23.0	15.2–30.8	28.6	18.9–38.2	34.0	20.5–47.6
>65	3.7	1.8–5.5	-	-	3.6	0–7.5	2.1	0–6.3
Education								
University	89.0	86.0–92.0	90.3	84.8–95.7	82.1	74.0–90.3	87.2	77.7–96.8
Non-academic	11.0	8.0–14.0	9.7	4.3–15.2	17.9	9.7–26.0	12.8	3.2–22.3
Taste								
Like milk better than plant-based beverages	77.8	73.7–81.8	58.4	49.3–67.5	22.6	13.7–31.6	31.9	18.6–45.2
Do not like milk better than plant-based beverages	22.2	18.2–26.4	41.6	32.5–50.7	77.4	68.4–86.3	68.1	54.8–81.4

^1^ DC = dairy consumers not feeling discomfort after drinking milk; DC-D = dairy consumers feeling discomfort after drinking milk; CEM = dairy consumers excluding milk not feeling discomfort after drinking milk; CEM-D = dairy consumers excluding milk feeling discomfort after drinking milk.

**Table 3:** Relative frequency and range (95% CI) of ‘I don’t know’ in the Likert-type questions by cluster ^1^.

Questions	DC (*n* = 409)	DC-D (*n* = 113)	CEM (*n* = 84)	CEM-D (*n* = 47)
Section B: Opinion on milk as a source of nutrients				
Q_B1_: Milk is an important source of calcium, phosphorus, magnesium, and vitamins	3.4 (1.7–5.2)	8.0 (3.0–13.0)	4.8 (0.2–9.3)	4.3 (0–10.0)
Q_B2_: Milk contains high-quality proteins	6.8 (4.4–9.3)	14.2 (7.7–20.6)	9.5 (3.2–15.8)	8.5 (0.5–16.5)
Q_B3_: Milk is a basic food, very complete, and relatively cheap	0.5 (0–1.2)	0.9 (0–2.6)	1.2 (0–3.5)	2.1 (0–6.3)
Q_B4_: Milk is a basic food during childhood	1.2 (0.2–2.3)	0.9 (0–2.6)	1.2 (0–3.5)	0
Q_B5_: Milk is a basic food during old age	7.8 (5.2–10.4)	4.4 (0.6–8.2)	14.3 (6.8–21.8)	6.4 (0–13.4)
Section C: Opinion on milk and health				
Q_C1_: Milk is a key food in the prevention of osteoporosis	8.1 (5.4–10.7)	15.0 (8.5–21.6)	13.1 (5.9–20.3)	6.4 (0–13.4)
Q_C2_: Milk consumption is related to a higher risk of obesity	14.7 (11.2–18.1)	20.4 (12.9–27.8)	19.0 (10.7–27.4)	23.4 (11.3–35.5)
Q_C3_: In adults, milk consumption is related to a greater risk of certain diseases such as diabetes or cardiovascular problems	19.8 (15.9–23.7)	30.1 (21.6–38.5)	28.6 (18.9–38.2)	34.0 (20.5–47.6)
Q_C4_: In children, milk consumption is linked to an increased risk of autism	14.4 (11.0–17.8)	23.0 (15.2–30.8)	29.8 (20.0–39.5)	27.7 (14.9–40.4)
Section D: Opinion on milk production and environmental impact				
Q_D1_: Dairy farms are the largest emitters of greenhouse gases	14.4 (11.0–17.8)	12.4 (6.3–18.5)	14.3 (6.8–21.8)	17.0 (6.3–27.8)
Q_D2_: Dairy farms pollute the environment more than most crops used to produce plant-based beverages	23.5 (19.4–27.6)	25.7 (17.6–33.7)	21.4 (12.7–30.2)	38.3 (24.4–52.2)
Q_D3_: I believe that it is important that farmers make efforts to improve the welfare of their animals	0.7 (0–1.6)	0.9 (0–2.6)	1.2 (0–3.5)	2.1 (0–6.3)
Q_D4_: Dairy farms are making great efforts to pollute less and be more sustainable	19.8 (15.9–23.7)	24.8 (16.8–32.7)	21.4 (12.7–30.2)	38.3 (24.4–52.2)

^1^ DC = dairy consumers not feeling discomfort after drinking milk; DC-D = dairy consumers feeling discomfort after drinking milk; CEM = dairy consumers excluding milk not feeling discomfort after drinking milk; CEM-D = dairy consumers excluding milk feeling discomfort after drinking milk.

**Table 4:** Section F questions regarding the opinion on A2 milk’s benefits and intention to purchase expressed as relative frequency (RF, %) and range (95% CI) by cluster ^1^.

Questions	DC (*n* = 409)		DC-D (*n* = 113)		CEM (*n* = 84)		CEM-D (*n* = 47)	
	RF	95% CI	RF	95% CI	RF	95% CI	RF	95% CI
Concerning A2 milk:								
Until now, I had never heard of A2 milk	79.0	75.0–82.9	83.2	76.3–90.1	91.7	85.8–97.6	74.5	62.0–86.9
Yes, I had heard about it, and I associate it with more natural milk	1.0	0–1.9	2.7	0–5.6	-	-	-	-
Yes, I had heard about it, and I associate it with healthier milk	9.0	6.3–11.8	5.3	1.2–9.4	4.8	0.2–9.3	19.1	7.9–30.4
Yes, I had heard about it, and I associate it with better-tasting milk	0.5	0–1.2	-	-	-	-	-	-
Yes, I had heard about it, and I associate it with more sustainable milk	0.7	0–1.6	2.7	0–5.6	1.2	0–3.5	-	-
Yes, I had heard about it, and I associate it with more expensive milk	4.6	2.6–6.7	1.8	0–4.2	1.2	0–3.5	4.3	0–10.0
Yes, I had heard about it, although I didn’t know what it was or relate it to anything	5.1	3.0–7.3	4.4	0.6–8.2	1.2	0–3.5	2.1	0–6.3
Considering its gastrointestinal benefits, do you think A2 milk could be a good option in your case?								
Yes	14.7	11.2–18.1	56.6	47.5–65.8	17.9	9.7–26.0	31.9	18.6–45.2
No	34.0	29.4–38.6	5.3	1.2–9.4	39.3	28.8–49.7	23.4	11.3–35.5
I don’t know	51.3	46.5–56.2	38.1	29.1–47.0	42.9	32.3–53.4	44.7	30.5–58.9
If you could easily find A2 dairy products in the supermarket, would you buy them?								
No, this product, in my case, is not relevant	56.7	51.9–61.5	18.6	11.4–25.8	64.3	54.0–74.5	44.7	30.5–58.9
Yes, but only if they had the same price as regular milk	26.2	21.9–30.4	44.2	35.1–53.4	20.2	11.6–28.8	23.4	11.3–35.5
Yes, even if they were 10% more expensive than regular milk	14.7	11.2–18.1	24.8	16.8–32.7	14.3	6.8–21.8	21.3	9.6–33.0
Yes, even if they were 20% more expensive than regular milk	0.7	0–1.6	8.0	3.0–13.0	1.2	0–3.5	6.4	0–13.4
I would be willing to pay even more than 20% of what regular milk costs	1.7	0.5–3.0	4.4	0.6–8.2	-	-	4.3	0–10.0

^1^ DC = dairy consumers not feeling discomfort after drinking milk; DC-D = dairy consumers feeling discomfort after drinking milk; CEM = dairy consumers excluding milk not feeling discomfort after drinking milk; CEM-D = dairy consumers excluding milk feeling discomfort after drinking milk.

## Data Availability

The original contributions presented in the study are included in the article, further inquiries can be directed to the corresponding author. The data presented in this study are available free of charge for any user at the official data repository CORA RDR (https://doi.org/10.34810/data2481).
